# Association of Obstructive Sleep Apnea Risk with Hypoxia-Inducible Factor Expression in Chronic Rhinosinusitis by Inflammatory Endotype

**DOI:** 10.3390/jcm15103873

**Published:** 2026-05-18

**Authors:** Hye Kyu Min, Sung-Wan Kim, Jin-Young Min

**Affiliations:** 1 Department of Otorhinolaryngology—Head and Neck Surgery, Kyung Hee University Hospital, Kyung Hee University College of Medicine, Seoul 02447, Republic of Korea; gprb9870@naver.com (H.K.M.); drkimsw@hanmail.net (S.-W.K.); 2 Department of Medicine, Graduate School, Kyung Hee University, Seoul 02447, Republic of Korea

**Keywords:** hypoxia-inducible factors, chronic rhinosinusitis, endotype, obstructive sleep apnea

## Abstract

**Background/Objectives**: Hypoxia and hypoxia-inducible factors (HIFs) have emerged as pivotal factors in the pathophysiology of chronic rhinosinusitis (CRS). Obstructive sleep apnea (OSA) may exacerbate hypoxia-driven sinonasal inflammation. This study evaluated the association between OSA risk and sinonasal HIF expression in CRS patients, focusing on the distinct profiles of eosinophilic (ECRS) and non-eosinophilic (NECRS) endotypes. **Methods**: Ethmoid mucosal tissues were collected from 64 CRS patients undergoing surgery. Patients were classified into ECRS or NECRS groups based on blood eosinophil counts, and into high- or low-risk OSA groups based on apnea-hypopnea index on polysomnography or sleep domain score from the 22-item sinonasal outcome test. Protein levels of HIF-1α, HIF-2α, and various inflammatory mediators were measured via multiplex immunoassay. **Results**: HIF-2α expression was significantly higher in the high-risk OSA group (116.80 ± 131.48 vs. 47.37 ± 42.14, *p* < 0.05), whereas HIF-1α levels were independent of OSA status. Following stratification by endotype, HIF-1α expression was significantly higher in NECRS than in ECRS (0.042 ± 0.020 vs. 0.034 ± 0.024, *p* < 0.05). Notably, high-risk OSA was associated with markedly increased HIF-2α only within the NECRS subgroup (115.52 ± 61.07 vs. 47.97 ± 31.03, *p* < 0.05). Correlation analyses demonstrated endotype-specific inflammatory coupling, showing that HIF-1α and HIF-2α were selectively linked to MMP-9 (r = 0.689, *p* < 0.05) and neutrophil-related markers (r = 0.925, *p* < 0.05) in NECRS while exhibiting broader cytokine correlations in ECRS (*p* < 0.05). **Conclusions**: Sinonasal HIF expression in CRS varies according to OSA risk and CRS endotype. OSA-associated hypoxic stress preferentially influences HIF-2α expression in NECRS, whereas this effect is attenuated in ECRS, likely due to the dominance of local type 2 inflammatory signaling.

## 1. Introduction

Chronic rhinosinusitis (CRS) represents a chronic inflammatory disorder affecting the mucosal lining of the nasal cavity and paranasal sinuses [1,2]. It substantially influences daily life and work performance, with an estimated prevalence of approximately 5–12% in the general population [1,2,3]. Recently, CRS was subdivided into eosinophilic (ECRS) and non-eosinophilic (NECRS) subtypes, which correlate with their inflammatory endotypes, which exhibit distinct inflammatory profiles and clinical characteristics. While ECRS is predominantly associated with type 2 inflammation mediated by eosinophils and Th2 cytokines, NECRS often exhibits a neutrophil-dominant pattern [1,2,4].

The sinonasal mucosa, functioning as the primary interface between the upper respiratory tract and the external environment, is particularly vulnerable to hypoxic conditions, and accumulating evidence indicates that such mucosal hypoxia plays a critical role in the pathophysiology and development of CRS [1,5,6]. In CRS, excessive tissue growth and polyp formation can exceed neovascular capacity, leading to local hypoxia. As mucosal thickening progresses and the sinuses become obstructed, oxygenation of the sinus cavity further declines, resulting in worsening tissue hypoxia [5,7].

A central mediator of hypoxic signaling is the hypoxia-inducible factor (HIF) family, which are transcription factors composed of an oxygen-responsive α-subunit and a constitutively expressed β-subunit. Three types of α-subunit, HIF-1α, HIF-2α, and HIF-3α, are currently known [8,9]. Formation of the HIF-α/HIF-β heterodimer is essential for binding to the hypoxia-responsive element (HRE), located within the promoters or enhancers of target genes [8]. In the nasal mucosa, HIF-1α and HIF-2α regulate a wide array of genes involved in angiogenesis, inflammation, and tissue remodeling [1,10,11]. Notably, HIF-1α contributes to nasal polyp formation in CRS by promoting epithelial barrier disruption and epithelial-to-mesenchymal transition [10,12,13]. In contrast, the specific biological functions of HIF-2α in the nasal mucosa is relatively poorly understood.

Obstructive sleep apnea (OSA) is a prototypical disorder associated with intermittent systemic hypoxia and oxidative stress, resulting from recurrent upper airway obstruction during sleep [14,15]. HIF-1α and HIF-2α also play distinct, reciprocal roles in mediating cellular and physiological responses to OSA-induced intermittent hypoxia (IH) [16]. Emerging studies suggest a potential bidirectional relationship between OSA and CRS, as both conditions share overlapping mechanisms of hypoxia-driven inflammation and mucosal remodeling [15,17,18]. However, the underlying mechanisms linking OSA-induced hypoxia to inflammatory responses in CRS have not been fully elucidated.

Given these findings, this study aimed to investigate whether HIFs serve as a potential mechanistic link between CRS and OSA by comparing HIF expression in CRS patients with different OSA statuses, and to further examine whether these expression patterns differ by CRS endotype in association with specific cytokine profiles.

## 2. Materials and Methods

### 2.1. Subjects

A total of 64 patients diagnosed with CRS who underwent endoscopic sinus surgery (ESS) at Kyung Hee University Hospital between March 2021 and January 2022 and between August 2022 and February 2024 were enrolled in this retrospective observational study using prospectively collected specimens and clinical data. The study consisted of sequential recruitment phases conducted under separate Institutional Review Board (IRB) oversight. The initial recruitment phase was approved under IRB protocol KHUH 2020-01-018, and additional approval/amendment for extended recruitment and subsequent analyses was obtained under protocol KHUH 2022-07-071 prior to continuation of enrollment.

Ethmoid mucosal samples were prospectively collected intraoperatively from patients with ECRS (n = 38) and NECRS (n = 26), and some analyses were subsequently performed retrospectively using the archived specimens and clinical data. ECRS was defined as peripheral blood eosinophil count > 250 cells/µL based on the European position paper on rhinosinusitis and nasal polyps guidelines [2]. The high-risk OSA group (n = 52) included patients who reported habitual snoring and had either an apnea–hypopnea index (AHI) > 5 events/hour on level I polysomnography (PSG) or sleep domain score from the 22-item sinonasal outcome test (Sleep-SNOT) > 17.5 [19].

The exclusion criteria were as follows: (1) age under 18 years; (2) use of antibiotics, systemic/topical corticosteroids, or other immunomodulatory agents within 4 weeks before surgery; (3) presence of fungal sinusitis, sinonasal tumors, or (4) a prior history of head and neck malignancy or radiation therapy. Written informed consent was obtained from all subjects involved in the study.

### 2.2. Tissue Preparation and Protein Analysis

Ethmoid mucosal tissue specimens obtained during ESS were finely minced with sterile scissors immediately after surgery. Each sample (approximately 10–20 mg) was homogenized in 600 μL of PRO-PREP™ protein extraction solution (iNtRON Biotechnology, Boston, MA, USA) and cell lysis was induced by incubation at –20 °C for 20–30 min. The lysates were then centrifuged at 16,000 × g for 4 min immediately prior to analysis or dilution (at least two-fold). The supernatants were collected for quantitative protein analysis. Total protein concentrations of each sample were determined using a standard protein assay, and all cytokine and HIF levels were normalized to total protein content and expressed as pg/mg protein. Concentrations of HIFs (HIF-1α, HIF-2α) and cytokines—including IL-1β, IL-6, IL-8, IL-10, IL-13, IL-17A, matrix metalloproteinase (MMP)-9, transforming growth factor (TGF)-β1, vascular endothelial growth factor (VEGF)-A, chemokine (C-X-C motif) ligand (CXCL)-1, periostin, human neutrophil elastase (ELA2/HNE), and eosinophil cationic protein (ECP)—were measured using the Human Premixed Multi-Analyte Kit and TGF-β Premixed Kit (Luminex^®^ Performance Assay, LXSAHM and FCSTM17; R&D Systems, Minneapolis, MN, USA).

Tissue eosinophils were counted on hematoxylin and eosin (H&E)-stained sections. For each specimen, areas of maximal eosinophilic infiltration (“hot spots”) were first identified at low magnification (×100). Eosinophils were then counted manually in three non-overlapping high-power fields (HPFs) at ×400 magnification, and the mean eosinophil count per HPF was recorded.

### 2.3. Preoperative Evaluation

Preoperative assessments included evaluation of CRS severity (Lund–Mackay [LM] and Lund–Kennedy [LK] scores), olfactory function (Korean version of the Sniffin’ sticks test II [KVSS II]), and subjective symptoms (SNOT-22). Allergic status was evaluated via skin prick or ImmunoCAP tests. We also reviewed medical records for demographics, comorbidities, surgical history (revision surgery), and laboratory findings (serum eosinophil counts, ECP, and total IgE) obtained within one month before surgery.

### 2.4. Evaluation of OSA Status

Eleven patients completed full-night, level I PSG in a sleep laboratory and were diagnosed with OSA, and they were subsequently classified into the high-risk OSA group. PSG data were manually scored by a certified sleep technician in accordance with the 2012 American Academy of Sleep Medicine scoring criteria.

The SNOT-22, a validated quality-of-life questionnaire for CRS, was used to evaluate symptom severity on a six-point scale ranging from 0 (none) to 5 (severe) for each question. The Sleep-SNOT subset includes eight items and yields a maximum score of 40. (Table 1) Forty-one patients with a Sleep-SNOT score ≥ 17.5 were also classified into the high-risk OSA group, while 12 patients with a score < 17.5 were classified into the low-risk OSA group [19]. Sleep-SNOT is not diagnostic and reflects symptom burden rather than confirmed sleep-disordered breathing.

### 2.5. Statistical Analysis

All statistical analyses were performed with SPSS software (version 26.0; IBM Corp., Armonk, NY, USA). Between-group comparisons were conducted using the Mann–Whitney U test for continuous variables and the chi-square test or Fisher’s exact test for categorical variables, as appropriate. Correlations between variables were assessed using Spearman’s rank correlation coefficient. No formal correction for multiple comparisons (e.g., Bonferroni or FDR) was applied due to exploratory nature and limited sample size. Statistical significance was determined using two-tailed tests with a threshold of a *p*-value of 0.05. Effect sizes were reported as mean differences with 95% confidence intervals for group comparisons. Given the exploratory nature of this study and the relatively small sample size, particularly in subgroup analyses, effect sizes are reported as correlation coefficients (Spearman’s rho).

## 3. Results

### 3.1. Patient Demographics

Table 2 describes demographic information in detail. There were no significant differences between the NECRS and ECRS groups in terms of age; sex; body mass index (BMI); and diabetes mellitus (DM), allergy and revision surgery statuses (*p* > 0.05 for all). The prevalence of hypertension (HTN) was significantly higher in the NECRS group than in the ECRS group (*p* = 0.013). The frequency of asthma, as well as the levels of tissue eosinophils, blood eosinophils and ECP—markers associated with type 2 inflammation—were significantly higher in the ECRS group compared to the NECRS group (*p* < 0.05 for all). LM score, SNOT-22 and sleep SNOT were significantly higher in the ECRS group, indicating greater disease extent and symptom burden (*p* < 0.05 for all).

Among patients with NECRS, those in the high-risk OSA group were significantly younger than those in the low-risk OSA group (46.90 ± 17.92 vs. 63.67 ± 6.86 years, *p* = 0.019). Tissue eosinophil counts, blood eosinophil counts, serum ECP, IgE, LK score, LM score and KVSS II score did not significantly differ. However, symptom burden measured by SNOT-22 and Sleep-SNOT was markedly greater in the high-risk OSA group (49.35 ± 20.10 vs. 8.50 ± 8.57, 21.40 ± 7.19 vs. 0.17 ± 0.41, *p* < 0.05 for all). A similar pattern was observed in the ECRS group: no significant differences were found between the high- and low-risk OSA groups regarding tissue and blood eosinophil counts, serum ECP, total IgE, and clinical severity (LK, LM, and KVSS II score). However, both SNOT-22 and Sleep-SNOT scores were substantially higher in the high-risk OSA group (58.32 ± 20.82 vs. 19.73 ± 10.65 and 26.22 ± 7.60 vs. 3.00 ± 3.69, respectively; all *p* < 0.05).

### 3.2. Expression of HIF-1α, HIF-2α, and Cytokines by OSA Status

Expression of HIF-2α was significantly elevated in the high-risk OSA group compared with low-risk OSA group (116.80 ± 131.48 vs. 47.37 ± 42.14, *p* = 0.028). In contrast, no significant differences were observed between the groups for HIF-1α or any other measured inflammatory mediators, including ELA2/HNE, ECP, VEGF-A, TGF-β1, MMP-9, periostin, CXCL1, and various interleukins (IL-1β, 6, 8, 10, 13, 17A) (all *p* > 0.05; Table 3).

### 3.3. Expression of HIF-1α, HIF-2α, and Cytokines in NECRS and ECRS by OSA Status

HIF-1α expression was significantly higher in the NECRS group than in the ECRS group (0.042 ± 0.020 vs. 0.034 ± 0.024, *p* = 0.049). Conversely, levels of VEGF-A, IL-6, IL-10, and IL-17A were significantly elevated in ECRS compared to NECRS (all *p* < 0.05). When analyzed by endotype, the impact of OSA status on HIF expression exhibited distinct patterns. Within the NECRS group, HIF-2α expression was significantly elevated in the high-risk OSA group compared with low-risk OSA group (115.52 ± 61.07 vs. 47.97 ± 31.03, *p* = 0.027). However, this difference was not significant in the ECRS subgroup (117.49 ± 157.97 vs. 46.89 ± 53.19, *p* = 0.157). Regardless of CRS endotype, no significant differences were observed between the high- and low-risk OSA groups in the expression of HIF-1α or other measured inflammatory mediators (all *p* > 0.05; Table 4).

### 3.4. Correlation of HIF-1α and HIF-2α with Cytokines in the High-Risk OSA Group by CRS Endotype

Spearman correlation analysis demonstrated distinct relationships between HIFs and cytokines in the sinonasal mucosa and CRS endotype in the high-risk OSA group. In the NECRS subgroup, HIF-1α expression only showed a significant positive correlation with MMP-9 (r = 0.689, 95% CI: 0.36 to 0.86; *p* = 0.004), whereas HIF-2α exhibited strong correlations with ELA2/HNE (r = 0.925, 95% CI: 0.81 to 0.97; *p* < 0.001) and ECP (r = 0.979, 95% CI: 0.94 to 0.99; *p* < 0.001).

By contrast, the ECRS subgroup displayed broader correlation profiles; HIF-1α was associated with HIF-2α (r = 0.521, 95% CI: 0.21 to 0.73; *p* = 0.006), ELA2/HNE (r = 0.612, 95% CI: 0.34 to 0.79; *p* = 0.002), ECP (r = 0.448, 95% CI: 0.12 to 0.69; *p* = 0.037), IL-10 (r = 0.486, 95% CI: 0.17 to 0.72; *p* = 0.007), IL-1β (r = 0.499, 95% CI: 0.19 to 0.73; *p* = 0.006), and IL-13 (r = 0.481, 95% CI: 0.16 to 0.72; *p* = 0.015), while HIF-2α demonstrated robust associations with HIF-1α (r = 0.521, 95% CI: 0.21 to 0.73; *p* = 0.006), ELA2/HNE (r = 0.974, 95% CI: 0.94 to 0.99; *p* < 0.001), ECP (r = 0.961, 95% CI: 0.92 to 0.98; *p* < 0.001), VEGF-A (r = 0.542, 95% CI: 0.24 to 0.75; *p* = 0.003), TGF-β1 (r = –0.517, 95% CI: –0.73 to –0.20; *p* = 0.005), and IL-10 (r = 0.515, 95% CI: 0.20 to 0.73; *p* = 0.005; Table 5). 

## 4. Discussion

In this study, we demonstrate the endotype-specific associations between OSA-related hypoxia and sinonasal HIF and inflammatory mediator expression. To the best of our knowledge, this is the first study to investigate these relationships, suggesting that HIF dysregulation may serve as a shared biological pathway through which hypoxic stress modulates sinonasal inflammation in CRS.

While the link between OSA and CRS is not yet fully elucidated, accumulating evidence suggests that the intermittent hypoxia and systemic inflammatory responses characteristic of OSA may contribute to sustained upper airway inflammation. A population-based retrospective cohort study reported that patients with OSA had a 3.18-fold higher risk of developing subsequent CRS compared with those without OSA [15]. Consistently, a study conducted in the United States demonstrated that patients with OSA exhibited a high prevalence of nasal mucosal inflammation, characterized by increased inflammatory cell infiltration and elevated levels of pro-inflammatory mediators [17].

HIFs are key molecular regulators that sense oxygen levels and coordinate adaptive responses to hypoxia by controlling genes involved in tissue development, inflammatory signaling, angiogenesis, erythropoiesis, cell survival and apoptosis, and metabolic reprogramming [8,20]. Dysregulation of HIF activity disrupts oxygen homeostasis and contributes to pathological conditions, such as tumorigenesis, ischemic vascular disorders, and immune-mediated inflammatory diseases [8,21]. HIF-1α and HIF-2α both regulate cellular responses to hypoxia but have distinct roles and expression patterns. HIF-1α is broadly expressed across a wide range of adult mammalian tissues, whereas HIF-2 is more restricted to specialized cell types including endothelial cells, hepatocytes, intestinal and pancreatic epithelial cells, and alveolar epithelial cells [8,22,23]. HIF-1α is typically induced during acute hypoxia and promotes the expression of genes associated with glycolysis, proinflammatory cytokines, and neutrophil activation, whereas HIF-2α is preferentially expressed under chronic or systemic hypoxic conditions and regulates angiogenic and immunomodulatory genes such as VEGF and IL-10 [8,21,24].

Previous studies examining hypoxia-related pathways in CRS have predominantly focused on nasal polyp tissue [10,12,13,25]. Several studies have reported increased HIF-1α expression in nasal polyps compared with non-polyp nasal mucosa, and transcriptomic analyses have revealed upregulation of hypoxia- and angiogenesis-related pathways in polyp specimens [10,12,26]. These findings suggest that nasal polyps reside in a relatively hypoxic microenvironment, potentially driven by tissue edema, remodeling, and impaired ventilation within obstructed sinus cavities [1,7]. However, investigations specifically addressing HIF-2α in nasal polyp tissue are limited and inconsistent, making it difficult to delineate the distinct regulatory patterns of individual HIF isoforms within polyp-associated inflammation [12]. Importantly, nasal polyps themselves represent a profoundly altered inflammatory niche characterized by dense eosinophilic or neutrophilic infiltration, basement membrane thickening, glandular hyperplasia, and extensive extracellular matrix remodeling [27,28]. Such exaggerated local inflammation may mask the impact of systemic hypoxia seen in OSA. Therefore, we assessed HIF expression in ethmoid mucosa, which is inflamed in CRS but less influenced by extreme local inflammatory and structural alterations.

In this study, we demonstrated that HIFs, particularly HIF-2α, may contribute to the pathophysiological interplay between CRS and OSA. While previous research has implicated mucosal hypoxia as a key driver of sinonasal inflammation and tissue remodeling in CRS, the present findings extend this concept by showing that OSA-related hypoxic stress is associated with altered expression of hypoxia-sensing molecules within the sinonasal mucosa. Notably, HIF-2α expression was significantly elevated in the high-risk OSA group, whereas HIF-1α levels did not differ according to OSA status, suggesting isoform-specific activation in response to OSA-associated hypoxic burden. However, while hypoxia-related pathways are clearly implicated in OSA, evidence specifically comparing HIF-1α and HIF-2α—especially in sinonasal tissue—is scarce, and further studies are needed to clarify their differential roles.

When CRS was stratified by endotype, distinct differences in HIF regulation were observed. HIF-1α expression was higher in NECRS than in ECRS, consistent with prior reports linking HIF-1α to neutrophilic inflammation and hypoxia-driven neutrophil persistence [25,26]. Hypoxia primarily induces neutrophilic inflammation by promoting HIF-1α expression, which leads to peri-airway neutrophil aggregation [1,29]. In addition, HIF-1α contributes to the persistence of neutrophilic inflammation by mediating hypoxia-induced inhibition of neutrophil apoptosis [30,31]. Furthermore, high-risk OSA was associated with increased HIF-2α expression in NECRS but not in ECRS, suggesting greater susceptibility of neutrophil-dominant CRS to systemic hypoxia. Previous studies have shown that HIF-2α, unlike HIF-1α, is preferentially activated under chronic or systemic hypoxic conditions, such as those observed in OSA, and regulates genes involved in angiogenesis, oxidative metabolism, and immunomodulation rather than acute proinflammatory responses [8,16,32]. In ECRS, the type 2-dominant inflammatory milieu may overshadow the effect of systemic or intermittent hypoxia on HIF expression, suggesting that hypoxic responses vary according to the local inflammatory microenvironment.

In NECRS, HIF-2α correlated strongly with neutrophil markers, particularly ELA2/HNE, suggesting predominantly canonical oxygen-dependent regulation through hypoxia-mediated stabilization [33,34]. HIF-2α stability is tightly controlled by oxygen-sensing prolyl hydroxylase domain enzymes (PHDs) and factor-inhibiting HIF (FIH), which hydroxylate HIF-α subunits under normoxic conditions to promote von Hippel–Lindau-mediated ubiquitination and proteasomal degradation, whereas hypoxia suppresses PHD/FIH activity and thereby permits HIF-2α accumulation and the transcription of hypoxia-responsive genes [8,35,36]. Thus, systemic or intermittent hypoxia from high-risk OSA may exert a more direct influence on mucosal HIF regulation in NECRS, allowing oxygen-dependent mechanisms to predominate.

**Figure 1:**
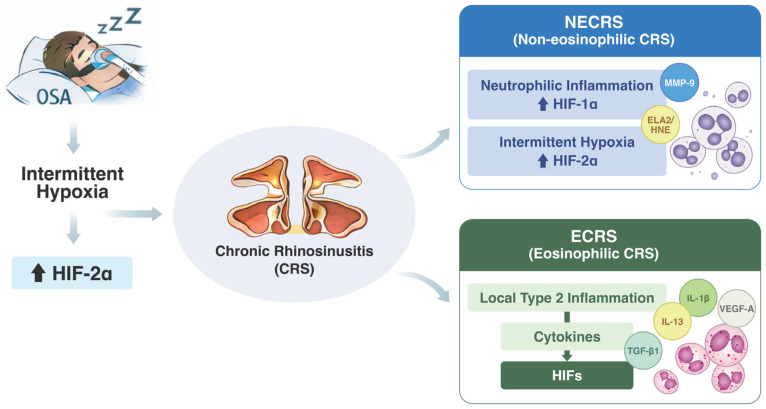
Proposed Model of OSA-Associated HIF Expression Across Inflammatory Endotypes in Chronic Rhinosinusitis.

In contrast, ECRS demonstrated a substantially broader and more complex pattern of associations between HIF isoforms and multiple inflammatory and remodeling mediators. This expanded correlation profile suggests that, in ECRS, HIF expression is predominantly modulated through oxygen-independent regulatory mechanisms rather than direct hypoxic stabilization alone. Numerous cytokines enriched in eosinophilic inflammation—such as IL-4 and IL-13 from Th2 cells, IL-1β and IL-6 from activated epithelial or myeloid cells, and IL-17 from Th17 responses—have been shown to transcriptionally or post-translationally upregulate HIF-1α or HIF-2α through pathways such as STAT6, NF-κB, ERK, PI3K/AKT, and mTOR signaling [25,37,38,39]. Within the highly inflamed, Th2-dominant microenvironment characteristic of ECRS, these non-hypoxic influences likely exert a cumulative effect that enhances or maintains HIF-1α and HIF-2α expression regardless of the prevailing oxygen tension [39,40,41]. In ECRS, cytokine- and metabolism-driven mechanisms may predominate in HIF regulation, thereby diminishing the impact of OSA-related intermittent hypoxia compared with NECRS. However, this remains a hypothesis requiring validation in human sinonasal models. The schematic illustration (Figure 1) summarizes the hypothetical model and key findings of the present study.

This study has several limitations. First, the identification of OSA risk relied primarily on the Sleep-SNOT questionnaire rather than gold-standard PSG for all patients, which may have limited the accuracy of our OSA classification. The use of questionnaire-based classification may not accurately reflect intermittent hypoxia and substantially limits mechanistic inference; therefore, future studies incorporating PSG-based diagnosis and objective parameters—such as CT90 (cumulative percentage of the time spent at saturations below 90%), lowest oxygen saturation, or hypoxic burden—are essential to clarify the dose–response relationship between OSA severity and HIF expression. Second, the cross-sectional design precludes establishing a definitive causal link between intermittent hypoxia and mucosal HIF dysregulation. Moreover, without evaluating upstream oxygen-sensing regulators such as PHD and FIH, the present study cannot disentangle the relative contributions of oxygen-dependent versus oxygen-independent mechanisms in modulating HIF signaling. Lastly, the relatively small sample size, particularly in the subgroup analyses, may have limited the statistical power to detect subtle differences in cytokine profiles or capture the full biological heterogeneity of CRS–OSA interactions. Future studies using prospective designs, PSG-confirmed OSA diagnosis, comprehensive assessments of hypoxic severity, and functional assays of HIF-related pathways will be valuable in elucidating the mechanistic interactions between OSA-induced hypoxia and endotype-specific inflammation in CRS. Despite these limitations, our findings provide a novel foundation for understanding the mechanistic interplay between systemic hypoxia and endotype-specific inflammation in CRS.

Taken together, these findings suggest that HIF signaling acts as a context-dependent regulator within the sinonasal mucosa, with oxygen-dependent mechanisms predominating in NECRS and oxygen-independent, cytokine-driven mechanisms exerting greater influence in ECRS. This endotype-specific divergence offers a potential explanation for the heterogeneous impact of OSA on CRS and highlights the importance of considering both systemic hypoxic burden and local inflammatory milieu when evaluating disease behavior in patients with comorbid OSA and CRS.

## 5. Conclusions

HIF signaling, particularly HIF-2α, may represent a biological bridge linking OSA-related hypoxic stress with sinonasal inflammation in CRS. High-risk OSA was associated with increased HIF-2α expression in NECRS but not in ECRS, and endotype-specific cytokine correlation patterns indicate that the response to systemic hypoxia may differ depending on the local inflammatory microenvironment. These findings highlight the importance of considering both systemic hypoxia and local inflammatory milieu in CRS with comorbid OSA.

## Figures and Tables

**Table 1:** Sleep subdomain of the sinonasal outcomes-22 questionnaire (sleep-SNOT).

13. Difficulty falling asleep	0	1	2	3	4	5
14. Wake up at night	0	1	2	3	4	5
15. Lack of a good night’s sleep	0	1	2	3	4	5
16. Wake up tired	0	1	2	3	4	5
17. Fatigue	0	1	2	3	4	5
18. Reduced productivity	0	1	2	3	4	5
19. Reduced concentration	0	1	2	3	4	5
20. Frustrated/restless/irritable	0	1	2	3	4	5

**Table 2:** Clinical characteristics of the patients.

	NECRS (N = 26)				ECRS (N = 38)				NECRS vs. ECRS *p*-Value
	NECRS Total (n = 26)	High-Risk OSA (n = 20)	Low-Risk OSA (n = 6)	*p*-Value	ECRS Total (n = 38)	High-Risk OSA (n = 32)	Low-Risk OSA (n = 6)	*p*-Value	
Age	50.77 ± 17.47	46.90 ± 17.92	63.67 ± 6.86	0.019 *	43.50 ± 13.08	42.66 ± 12.68	48.00 ± 15.49	0.493	0.056
Sex (male)	17 (65.38)	12 (60.0)	5 (83.3)	0.292	26 (68.42)	23 (71.9)	3 (50.0)	0.290	0.799
BMI	25.15 ± 3.69	25.11 ± 3.82	25.27 ± 3.54	0.533	24.28 ± 3.12	24.28 ± 3.31	24.28 ± 2.08	0.830	0.397
Comorbidities									
HTN	13 (50)	7 (35.0)	6 (100)	0.005 *	7 (18.42)	5 (15.6)	2 (33.3)	0.305	0.013 *
DM	7 (26.92)	4 (20.0)	3 (50.0)	0.146	4 (10.53)	3 (9.4)	1 (16.7)	0.593	0.104
Asthma	3 (11.54)	3 (15.0)	0 (0.0)	0.313	15 (39.47)	10 (31.2)	5 (83.3)	0.017 *	0.022 *
Allergy	16 (61.54)	14 (70.0)	2 (33.3)	0.105	18 (47.37)	15 (46.9)	3 (50.0)	0.888	0.314
Revision surgery	1 (3.85)	1 (5.0)	0 (0.0)	0.576	3 (7.89)	3 (9.4)	0 (0.0)	0.435	0.640
Tissue Eo	26.88 ± 33.59	31.00 ± 39.75	20.00 ± 25.98	0.571	102.81 ± 65.78	94.23 ± 67.21	140.00 ± 52.92	0.439	0.000 *
Blood_Eo	129.57 ± 62.79	125.56 ± 56.87	142.91 ± 84.54	0.700	627.34 ± 600.30	626.95 ± 645.05	629.46 ± 294.84	0.422	0.000 *
Blood_ECP	14.73 ± 11.30	13.87 ± 11.89	17.45 ± 9.59	0.121	47.25 ± 40.24	47.46 ± 41.72	46.20 ± 35.21	0.820	0.000 *
Blood_IgE	218.02 ± 410.01	257.52 ± 465.08	99.51 ± 124.14	0.280	488.50 ± 1410.01	510.83 ± 1538.16	376.83 ± 420.22	0.467	0.143
LK_score	4.27 ± 3.04	4.45 ± 3.17	3.67 ± 2.73	0.494	5.37 ± 2.80	5.13 ± 2.73	6.67 ± 3.08	0.297	0.078
LM_score	9.39 ± 6.60	9.25 ± 6.65	9.83 ± 7.03	0.700	14.29 ± 7.05	14.59 ± 7.26	12.67 ± 6.06	0.598	0.005 *
KVSS II	31.89 ± 11.88	32.38 ± 12.42	30.25 ± 10.73	0.219	26.15 ± 12.40	25.74 ± 13.00	28.33 ± 9.09	0.953	0.054
SNOT-22	39.92 ± 25.10	49.35 ± 20.10	8.50 ± 8.57	0.000 *	53.68 ± 23.81	58.32 ± 20.82	19.73 ± 10.65	0.000 *	0.028 *
Sleep-SNOT	16.50 ± 11.07	21.40 ± 7.19	0.17 ± 0.41	0.000 *	22.55 ± 11.13	26.22 ± 7.60	3.00 ± 3.69	0.000 *	0.010 *

NECRS, Non-Eosinophilic Chronic RhinoSinusitis; ECRS, Eosinophilic Chronic RhinoSinusitis; OSA, Obstructive sleep apnea; BMI, Body Mass Index; HTN, Hypertension; DM, Diabetes Mellius; Tissue Eo, Tissue Eosinophil count; Blood Eo, Blood Eosinophil count; ECP, Eosinophil Cationic Protein; LK, Lund-Kennedy; LM. Lund-Mackay; KVSS II, Korean version of the Sniffin’ Sticks test II; SNOT-22, 22-item Sinonasal Outcome Test; Sleep-SNOT, Sleep domain score from the 22-item Sinonasal Outcome Test; * *p* < 0.05.

**Table 3:** Expression of HIF-1α, HIF-2α, and cytokines by OSA status.

	Total (N = 64)	High-Risk OSA (n = 52)	Low-Risk OSA (n = 12)	*p*-Value
HIF-1α	0.037 ± 0.022	0.038 ± 0.022	0.035 ± 0.027	0.511
HIF-2α	104.79 ± 123.36	116.80 ± 131.48	47.37 ± 42.14	0.028 *
ELA2/HNE	606.14 ± 752.26	661.30 ± 803.61	309.65 ± 219.57	0.101
ECP	353.36 ± 382.98	371.60 ± 404.50	225.64 ± 120.91	0.512
VEGF-A	72.57 ± 53.63	71.50 ± 52.53	77.10 ± 60.34	0.986
TGF-ß1	1645.67 ± 1223.99	1606.50 ± 1000.92	1827.24 ± 2021.22	0.549
MMP-9	11,609.06 ± 17,625.30	12,693.06 ± 19,576.08	7544.08 ± 5076.66	0.754
IL-6	18.81 ± 50.17	21.42 ± 55.02	6.73 ± 4.68	0.227
IL-8	209.75 ± 428.04	230.75 ± 458.21	83.73 ± 93.41	0.315
IL-10	5.09 ± 3.69	5.10 ± 3.84	5.03 ± 3.12	0.891
IL-1ß	12.75 ± 16.51	13.29 ± 18.16	10.46 ± 5.40	0.484
IL-17A	12.66 ± 9.49	12.96 ± 10.27	11.50 ± 5.49	0.839
IL-13	307.75 ± 223.19	317.03 ± 231.73	271.50 ± 191.48	0.644
Periostin	254,967.13 ± 193,394.89	260,209.71 ± 179,980.99	233,996.79 ± 248,196.88	0.144
CXCL1	1431.95 ± 1922.11	1468.12 ± 1878.57	1267.53 ± 2199.69	0.107

OSA, Obstructive sleep apnea; HIF, Hypoxia-inducible factor; ELA2/HNE, Human neutrophil elastase; ECP, Eosinophil cationic protein; VEGF, Vascular endothelial growth factor; TGF, Transforming growth factor; MMP, Matrix metalloproteinase; IL, Interleukin; CXCL, Chemokine (C-X-C motif) ligand; * *p* < 0.05.

**Table 4:** Expression of HIF-1α, HIF-2α, and cytokines in NECRS and ECRS by OSA status.

	NECRS (N = 26)				ECRS (N = 38)				NECRS vs. ECRS *p*-Value
	NECRS Total (n = 26)	High-Risk OSA (n = 20)	Low-Risk OSA (n = 6)	*p*-Value	ECRS Total (n = 38)	High-Risk OSA (n = 32)	Low-Risk OSA (n = 6)	*p*-Value	
HIF-1α	0.042 ± 0.020	0.040 ± 0.017	0.046 ± 0.031	0.971	0.034 ± 0.024	0.036 ± 0.025	0.021 ± 0.010	0.262	0.049 *
HIF-2α	101.30 ± 62.14	115.52 ± 61.07	47.97 ± 31.03	0.027 *	106.79 ± 148.56	117.49 ± 157.97	46.89 ± 53.19	0.157	0.157
ELA2/HNE	465.49 ± 301.33	508.79 ± 311.45	270.66 ± 155.19	0.141	712.85 ± 956.61	771.12 ± 1015.59	348.65 ± 290.44	0.374	0.732
ECP	261.87 ± 160.69	283.08 ± 167.28	166.45 ± 85.63	0.166	430.77 ± 490.36	438.00 ± 510.16	344.01 ± 95.19	0.498	0.374
VEGF-A	58.31 ± 49.94	61.53 ± 55.93	47.58 ± 19.99	0.882	82.59 ± 54.52	77.93 ± 50.09	106.63 ± 74.28	0.456	0.002 *
TGF-ß1	1726.43 ± 1194.49	1871.10 ± 1200.06	1244.21 ± 1139.63	0.219	1587.34 ± 1258.40	1435.80 ± 825.15	2526.89 ± 2732.79	0.657	0.578
MMP-9	13,124.23 ± 20,639.31	15,128.16 ± 23,689.71	7446.43 ± 5111.82	0.708	10,584.10 ± 15,507.93	11,214.61 ± 16,909.95	7641.72 ± 5526.83	0.982	0.603
IL-6	11.81 ± 23.59	14.08 ± 26.59	4.25 ± 2.75	0.242	23.87 ± 62.66	26.16 ± 67.37	9.70 ± 5.01	0.929	0.013 *
IL-8	268.92 ± 596.42	310.71 ± 661.18	101.78 ± 118.79	0.408	162.03 ± 214.63	173.65 ± 223.00	53.64 ± 12.13	0.826	0.570
IL-10	4.07 ± 3.93	4.14 ± 4.39	3.85 ± 1.96	0.656	5.79 ± 3.10	5.71 ± 3.39	6.22 ± 3.77	0.740	0.003 *
IL-1ß	13.09 ± 22.45	14.12 ± 25.59	9.63 ± 3.72	0.242	12.51 ± 10.91	12.75 ± 11.59	11.29 ± 6.97	0.857	0.230
IL-17A	10.81 ± 10.77	11.13 ± 12.03	9.81 ± 5.79	0.642	13.99 ± 8.36	14.15 ± 8.96	13.20 ± 5.08	0.848	0.026 *
IL-13	268.90 ± 267.29	276.02 ± 298.95	249.93 ± 177.11	0.858	334.46 ± 186.98	341.33 ± 183.14	297.37 ± 255.64	0.650	0.060
Periostin	217,189.32 ± 179,699.41	232,486.80 ± 202,686.70	168,747.28 ± 58,317.85	0.642	281,951.28 ± 200,772.34	278,373.00 ± 164,624.50	299,246.29 ± 349,148.61	0.379	0.092
CXCL1	1049.42 ± 1050.26	1172.14 ± 1148.58	640.35 ± 491.03	0.196	1716.11 ± 2348.11	1665.43 ± 2235.57	2020.15 ± 3239.92	0.477	0.610

NECRS, Non-Eosinophilic Chronic RhinoSinusitis; ECRS, Eosinophilic Chronic RhinoSinusitis; OSA, Obstructive sleep apnea; HIF, Hypoxia-inducible factor; ELA2/HNE, Human neutrophil elastase; ECP, Eosinophil cationic protein; VEGF, Vascular endothelial growth factor; TGF, Transforming growth factor; MMP, Matrix metalloproteinase; IL, Interleukin; CXCL, Chemokine (C-X-C motif) ligand; * *p* < 0.05.

**Table 5:** Correlation between HIF-1α, HIF-2α and cytokines and CRS endotype in the high-risk OSA group.

	NECRS/High-Risk OSA (N = 20)						ECRS/High-Risk OSA (N = 32)					
	HIF-1α			HIF-2α			HIF-1α			HIF-2α		
	Spearman	95% CI	*p*-Value	Spearman	95% CI	*p*-Value	Spearman	95% CI	*p*-Value	Spearman	95% CI	*p*-Value
HIF-1α				–0.095	–0.51 to 0.36	0.748				0.521	0.21 to 0.73	0.006 *
HIF-2α	–0.095	–0.51 to 0.36	0.748				0.521	0.21 to 0.73	0.006 *			
ELA2/HNE	–0.088	–0.50 to 0.37	0.736	0.925	0.81 to 0.97	0.000 *	0.612	0.34 to 0.79	0.002 *	0.974	0.94 to 0.99	0.000 *
ECP	–0.157	–0.55 to 0.31	0.548	0.979	0.94 to 0.99	0.000 *	0.448	0.12 to 0.69	0.037 *	0.961	0.92 to 0.98	0.000 *
VEGF-A	–0.024	–0.46 to 0.42	0.926	0.189	–0.27 to 0.58	0.499	0.212	–0.15 to 0.52	0.270	0.542	0.24 to 0.75	0.003 *
TGF-ß1	0.098	–0.36 to 0.52	0.699	−0.100	–0.51 to 0.36	0.723	0.037	–0.32 to 0.38	0.847	−0.517	–0.73 to –0.20	0.005 *
MMP-9	0.689	0.36 to 0.86	0.004 *	0.189	–0.27 to 0.58	0.557	−0.007	–0.36 to 0.35	0.971	−0.118	–0.44 to 0.24	0.575
IL-6	–0.292	–0.64 to 0.18	0.240	0.157	–0.30 to 0.56	0.576	0.017	–0.34 to 0.36	0.931	−0.287	–0.59 to 0.07	0.138
IL-8	–0.073	–0.49 to 0.38	0.773	–0.114	–0.52 to 0.35	0.685	−0.106	–0.43 to 0.26	0.605	−0.219	–0.53 to 0.14	0.292
IL-10	–0.218	–0.59 to 0.25	0.385	0.471	0.05 to 0.75	0.076	0.486	0.17 to 0.72	0.007 *	0.515	0.20 to 0.73	0.005 *
IL-1ß	0.063	–0.41 to 0.49	0.804	0.318	–0.15 to 0.66	0.248	0.499	0.19 to 0.73	0.006 *	0.233	–0.13 to 0.54	0.242
IL-17A	−0.319	–0.66 to 0.15	0.213	0.301	–0.17 to 0.65	0.296	0.297	–0.06 to 0.59	0.132	0.177	–0.18 to 0.49	0.377
IL-13	−0.061	–0.48 to 0.39	0.830	0.291	–0.18 to 0.64	0.334	0.481	0.16 to 0.72	0.015 *	0.389	0.05 to 0.65	0.054
Periostin	−0.328	–0.66 to 0.14	0.198	0.475	0.06 to 0.75	0.074	0.239	–0.11 to 0.54	0.230	0.021	–0.33 to 0.36	0.918
CXCL1	−0.350	–0.67 to 0.12	0.155	0.179	–0.28 to 0.57	0.524	−0.021	–0.36 to 0.32	0.914	0.085	–0.28 to 0.42	0.674

NECRS, Non-Eosinophilic Chronic RhinoSinusitis; ECRS, Eosinophilic Chronic RhinoSinusitis; OSA, Obstructive sleep apnea; HIF, Hypoxia-inducible factor; ELA2/HNE, Human neutrophil elastase; ECP, Eosinophil cationic protein; VEGF, Vascular endothelial growth factor; TGF, Transforming growth factor; MMP, Matrix metalloproteinase; IL, Interleukin; CXCL, Chemokine (C-X-C motif) ligand; * *p* < 0.05.

## Data Availability

The data are available from the corresponding author upon reasonable request.
